# Hypertension in Children with Obstructive Sleep Apnea Syndrome—Age, Weight Status, and Disease Severity

**DOI:** 10.3390/ijerph18189602

**Published:** 2021-09-12

**Authors:** Hai-Hua Chuang, Jen-Fu Hsu, Chao-Yung Wang, Li-Pang Chuang, Min-Chi Chen, Ning-Hung Chen, Yu-Shu Huang, Hsueh-Yu Li, Li-Ang Lee

**Affiliations:** 1 Department of Family Medicine, Chang Gung Memorial Hospital, Taipei Branch and Linkou Main Branch, Taoyuan 33305, Taiwan; chhaihua@gmail.com; 2 Department of Industrial Engineering and Management, National Taipei University of Technology, Taipei 10608, Taiwan; 3 Obesity Institute, Genomic Medicine Institute, Geisinger Health, Danville, PA 17822, USA; 4 College of Medicine, Chang Gung University, Taoyuan 33302, Taiwan; jeff0724@gmail.com (J.-F.H.); r5243@cgmh.org.tw (L.-P.C.); mcc@mail.cgu.edu.tw (M.-C.C.); ninghung@yahoo.com.tw (N.-H.C.); yushuhuang1212@gmail.com (Y.-S.H.); hyli38@cgmh.org.tw (H.-Y.L.); 5 Department of Pediatrics, Chang Gung Memorial Hospital, Linkou Main Branch, Taoyuan 33305, Taiwan; 6 Department of Cardiology, Chang Gung Memorial Hospital, Linkou Main Branch, Chang Gung University, Taoyuan 33305, Taiwan; cwang@ocean.ag; 7 Department of Pulmonary and Critical Care Medicine, Chang Gung Memorial Hospital, Linkou Main Branch, Taoyuan 33305, Taiwan; 8 Department of Public Health and Biostatistics, Consulting Center, Chang Gung University, Taoyuan 33302, Taiwan; 9 Department of Child Psychiatry, Chang Gung Memorial Hospital, Linkou Main Branch, Taoyuan 33305, Taiwan; 10 Department of Otorhinolaryngology-Head and Neck Surgery, Chang Gung Memorial Hospital, Linkou Main Branch, Taoyuan 33305, Taiwan

**Keywords:** age, apnea-hypopnea index, blood oxygen saturation, children, hypertension, obesity, obstructive sleep apnea syndrome

## Abstract

Older age, obesity, and obstructive sleep apnea syndrome (OSAS) are known to increase the risk of hypertension in adults. However, data for children are scarce. This study aimed to investigate the relationships between hypertension, age, weight status, and disease severity in 396 children with OSAS. The prevalence rates of hypertension, obesity, and severe OSAS (apnea-hypopnea index ≥10) were 27.0%, 28.0%, and 42.9%, respectively. Weight z-score and apnea-hypopnea index were independently correlated with systolic blood pressure z-score, and minimal blood oxygen saturation (SpO_2_) was independently associated with diastolic blood pressure z-score. Overall, late childhood/adolescence (odds ratio (OR) = 1.72, 95% CI = 1.05–2.81), obesity (OR, 2.58, 95% CI = 1.58–4.22), and severe OSAS (OR = 2.38, 95% CI = 1.48–3.81) were independent predictors of pediatric hypertension. Furthermore, late childhood/adolescence (OR = 2.50, 95% CI = 1.10–5.71) and abnormal SpO_2_ (mean SpO_2_ < 95%; OR = 4.91, 95% CI = 1.81–13.27) independently predicted hypertension in obese children, and severe OSAS (OR = 2.28, 95% CI = 1.27–4.10) independently predicted hypertension in non-obese children. In conclusion, obesity, OSAS severity, and abnormal SpO_2_ are potentially modifiable targets to improve hypertension while treating children with OSAS.

## 1. Introduction

Approximate 4–11% of children have obstructive sleep apnea syndrome (OSAS), characterized by abnormal respiration and ventilation during sleep [1]. OSAS encompasses a spectrum of disease severity, ranging from primary snoring to obstructive sleep apnea (OSA) [2]. Notably, even children with primary snoring have reduced attention and higher levels of social problems and anxiety/depressive symptoms than non-snoring children [3]. The prevalence of OSAS reaches a peak during preschool years due to adenotonsillar hypertrophy, which compromises the upper airway [4]. Risk factors for childhood OSA include increased neck circumference, tonsil size, adenoid grade, body mass index (BMI) z-score, and diastolic blood pressure (DBP) [5]. Although preschool-aged children with OSAS have less severe cardiovascular and neurocognitive problems [6], school-aged children with OSAS may have recognizable sequelae [7].

Hypertension is not an uncommon disease in children, with a reported global prevalence of 4% [8]. Traditional risk factors for pediatric hypertension include male sex, older age, obesity, high sodium intake, ethnicity (African American or Latino ancestry), family history of hypertension, and poor sleep quality [9,10]. OSAS is a known independent risk factor for hypertension in adults [11]. However, whether OSAS contributes to hypertension in children remains unclear. Nisbet et al. proposed that elevated sympathetic activity and impaired autonomic reflexes can lead to blood pressure (BP) dysregulation in school-aged children and adolescents with OSAS [12]. Brooks et al. demonstrated that breathing problems could disrupt sleep stages, and that a higher apnea-hypopnea index (AHI) was related to higher DBP and blunted BP changes during sleep, higher BP during rapid eye movement sleep, and systolic hypertension [13]. Notably, intermittent hypoxemia has been proposed as one of the possible pathogenic mechanisms involved in the BP elevation observed in OSA [14]. In addition, a large study of 700 randomly selected children found that OSAS, along with older age, BMI percentile, waist circumference, and snoring, were independently associated with an increase in BP [15]. A longitudinal study of 334 children also found that children with OSAS tended to have increased systolic BP (SBP) over a five-year period [16]. However, in contrast, a previous meta-analysis did not support that children with moderate-to-severe OSAS had an increased risk of elevated BP [17].

OSAS may worsen BP and arterial stiffness in obese children [18]. Thus, obesity is one of the most important risk factors for significant disease severity among children with OSAS [19], and those who have both conditions are at a greater risk of adverse outcomes of various organ systems. For example, Walter et al. reported that overweight/obese children with OSAS were more likely to have arterial stiffness than normal-weight children with OSAS and non-snoring controls [20]. Obesity has also been associated with exercise deconditioning in children with OSAS [21]. Moreover, pediatric OSA patients with different weight status have been shown to have different degrees of systemic inflammation [22].

Previous studies have observed relationships between OSAS, obesity, and hypertension in adults. However, the interactions between obesity and hypertension have not been elucidated among children with OSAS. Therefore, we hypothesized that, in this population, (1) obesity may independently contribute to pediatric hypertension, and (2) obesity may have an impact on the interactions between OSAS parameters and BP. Therefore, among a cohort of children with OSAS, the current study aimed to: (1) identify possible factors contributing to pediatric hypertension by examining the differences between demographic, anthropometric, and polysomnographic measures in those with and without hypertension, and (2) investigate how predictors of pediatric hypertension differed among the overall cohort and subgroups with and without obesity.

## 2. Materials and Methods

### 2.1. Study Design and Data Collection

In this retrospective cross-sectional study, data were retrieved from a dataset of pediatric patients with OSAS who were referred because of chronic loud snoring to the Department of Otorhinolaryngology, Head and Neck Surgery, Chang Gung Memorial Hospital, Linkou Main Branch (Taoyuan City, Taiwan) between 01 January 2010 and 31 December 2019. Medical chart reviews were performed from 01 June 2020 to 31 December 2020. The study was approved by the Institutional Review Board of the Chang Gung Memorial Foundation (protocol code: 202000873B0; date of approval: 22 May 2020). Since the current study was based on a secondary analysis of existing data, the requirement for written informed consent was waived. The STROBE guidelines were followed [23].

### 2.2. Patient Selection

**Figure 1:**
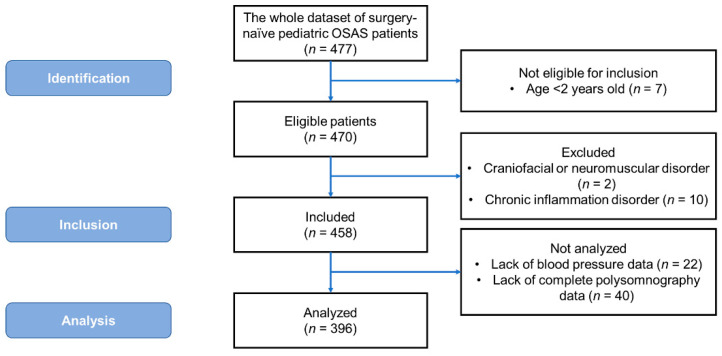
The Strengthening the Reporting of Observational Studies in Epidemiology (STROBE) [23] flow diagram of the study participants. Abbreviation: OSAS: obstructive sleep apnea syndrome.

A sample of 396 children diagnosed with OSAS from 477 consecutive eligible patients was included and analyzed in the study, as shown in Figure 1. OSAS was defined as frequent or loud snoring, nasal obstruction, mouth breathing, or witnessed breathing pauses during sleep [2,24]. OSA was defined as obstructive AHI ≥ 1 events/h [25]. The inclusion criteria were: (a) age range from 2 to 17 years; and (b) having standard polysomnography data at baseline. The exclusion criteria were: (a) craniofacial or neuromuscular disorders [25]; and (b) chronic inflammatory disorders such as asthma or autoimmune diseases [5]. In addition, patients without BP data and those without complete polysomnography data were not included for statistical analysis.

### 2.3. Measurements

#### 2.3.1. Demographic and Anthropometric Variables

In this study, age was categorized into three developmental stages (toddler/preschooler: 2 years ≤ age < 5 years; early childhood: 5 years ≤ age < 8 years; late childhood/adolescence: 8 years ≤ age < 18 years). Body height and body weight were measured. Age and sex adjusted z-scores of height, weight, and BMI were calculated according to the United States Centers for Disease Control and Prevention 2000 growth charts [26]. In this study, obesity was defined as a BMI z-score ≥ 1.645 [27]. Furthermore, a family history of hypertension, OSAS, or obesity was recorded.

#### 2.3.2. Polysomnography

Each participant received standard full-night in-laboratory polysomnography with a family member present to document the OSAS parameters [28]. Apnea was defined as ≥90% decrease in airflow for a duration of ≥2 breaths, and hypopnea was defined as ≥50% decrease in airflow and either ≥3% desaturation or electroencephalographic arousal for a duration of ≥2 breaths. The AHI was calculated by dividing the number of all apneas and hypopneas by the number of hours of sleep, according to the 2012 American Academy of Sleep Medicine Scoring Manual [29]. Polysomnographic data were reviewed, and the AHI, mean blood oxygen saturation (SpO_2_), and minimal SpO_2_ were recorded.

#### 2.3.3. Blood Pressure

BP was measured three times with a standard sphygmomanometer (Dinamap ProCare 100; GE Medical Systems Information Technologies, Inc., Milwaukee, WI, USA) between 10:00 and 11:00 PM, before the polysomnography. The detailed procedure of measuring BP has been described elsewhere [30]. Age, sex, and height-adjusted z-scores of SBP and DBP were calculated for each child. Pediatric hypertension was defined as an SBP z-score or DBP z-score ≥ 1.645 based on age, sex, and height z-score [31].

### 2.4. Outcome Variables and Covariates

The primary outcome variable was hypertension. Other outcome variables included SBP z-score, DBP z-score, BMI z-score, AHI, mean SpO_2_, and minimal SpO_2_. Clinically important variables, including developmental stage, obesity, disease severity, abnormal SpO_2_, and significant hypoxemia, were also analyzed. Covariates included age, sex, and height.

### 2.5. Statistical Analysis

Continuous data were compared using the independent-samples *t*-test. Levene’s test was performed to check if the datasets fulfill the homogeneity of variance assumption before the *t*-test was performed. If the variances of two imbalanced samples were equal, the Student’s *t*-test was performed. If the variances of two samples were unequal, Welch’s *t*-test was performed. Ordinal variables were compared using the Mantel–Haenszel test for trend, and nominal variables were compared using the chi-square test or Fisher’s exact test as appropriate.

The relationships between continuous variables were assessed using the Pearson correlation test, and the relationships between continuous, ordinal, and categorical variables were analyzed using the Spearman correlation test. To improve the regression performance, external information and the sign of regression coefficients with full models, including all clinical variables, were incorporated using multivariate linear or logistic regression models [32]. Furthermore, a parsimonious model was obtained using a forward selection approach with a significance level (alpha) of 0.10. All *p*-values were two-sided, and statistical significance was accepted at *p* < 0.05. All statistical analyses were performed using SPSS software (version 25; International Business Machines Corp., Armonk, NY, USA).

## 3. Results

### 3.1. Demographic, Anthropometric, Polysomnographic, and Blood Pressure Characteristics

The whole dataset included 477 surgery-naïve children with OSAS during the study period. Seven patients were not eligible for inclusion as they were aged <2 years. Among the 470 eligible patients, one patient with a craniofacial disorder, one patient with a neuromuscular disorder, and 10 patients with chronic inflammatory disorders were excluded. A further 22 patients who lacked BP data and 40 patients who lacked complete polysomnography data were also excluded from the analysis (Figure 1).

Herein, descriptive statistics included means (standard deviations) and numbers (proportions) and were used to characterize this population and evaluate distributions of the outcome variables. A total of 396 Taiwanese children with OSAS (114 (28.8%) girls and 282 (71.2%) boys) were included for analysis. The mean age was 7.50 (2.72) years. The mean weight z-score was 0.552 (1.497), the mean height z-score was 0.168 (1.161), and the mean BMI z-score was 0.607 (1.468). The mean AHI, mean SpO_2_, and minimal SpO_2_ were 15.32 (19.36) events/h, 95.78% (2.97%), and 85.43% (9.15%), respectively. In addition, the mean SBP z-score was 0.363 (1.526) and the mean DBP z-score was 0.651 (1.001). Fifty-three (13.4%) participants had a family history of hypertension, seven (1.8%) had a family history of OSAS, and five (1.3%) had a family history of obesity.

In this study, the participants were categorized into three OSAS disease severity subgroups: grade 1 (mild OSAS: AHI < 5 event/h), grade 2 (moderate OSAS: 5 events/h ≤ AHI < 10 events/h), and grade 3 (severe OSAS: AHI ≥ 10 events/h) [33,34]. Abnormal SpO_2_ was defined as mean SpO_2_ < 95% [29], and significant hypoxemia was defined as minimal SpO_2_ < 85% [35]. Notably, 107 (27.0%) had pediatric hypertension, 170 (42.9%) had severe OSAS, and 111 (28.0%) had obesity. In addition, 73 (18.4%) had abnormal SpO_2_, and 132 (33.3%) had significant hypoxemia.

### 3.2. Differences in Clinical Variables between the Hypertensive and Normotensive Groups

SBP z-score, DBP z-score, weight z-score, BMI z-score, and AHI in the hypertensive group were significantly higher than those in the normotensive group (all *p* < 0.05) (Table 1). In contrast, mean SpO_2_ and minimal SpO_2_ in the hypertensive group were significantly lower than those in the normotensive group (both *p* < 0.05). However, age and height z-scores of both groups were comparable (*p* = 0.115 and 0.064).

**Figure 2:**
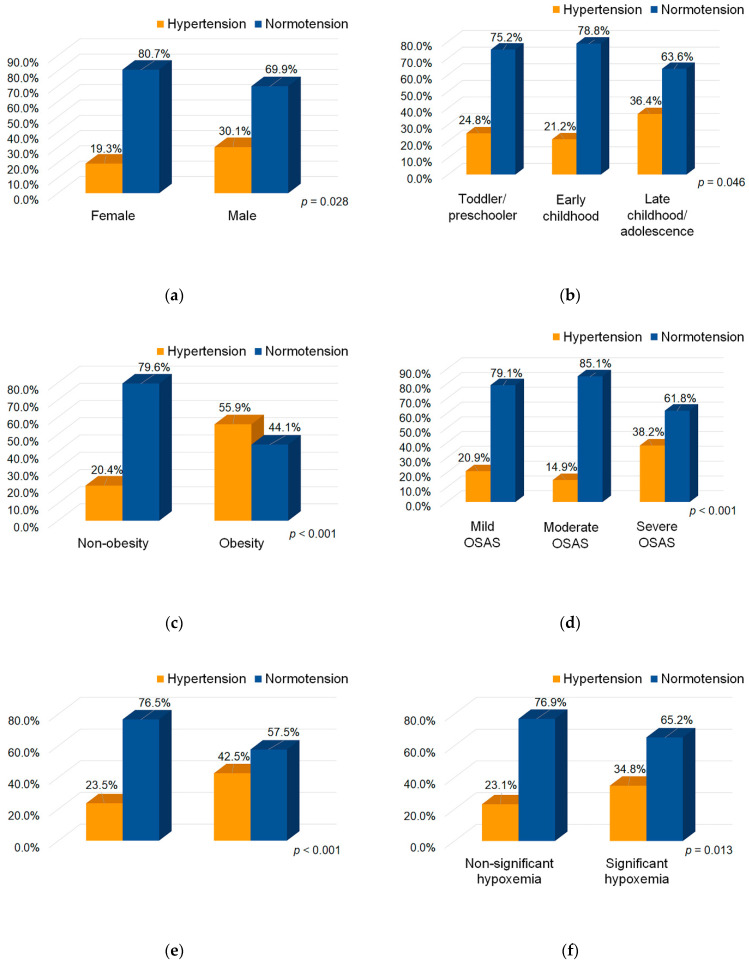
Bart charts demonstrate the relationships between hypertension and different clinical variable subgroups. (**a**) Sex subgroups; (**b**) Development stage subgroups; (**c**) Obesity subgroups; (**d**) Disease severity subgroups; (**e**) Abnormal oxygen saturation subgroups; (**f**) Significant hypoxemia subgroups. Abbreviations: OSAS: obstructive sleep apnea syndrome; SpO_2_: oxygen saturation.

The male patients had a significantly higher rate of hypertension than the female patients (*p* = 0.028) (Figure 2a), and a trend of an increasing prevalence of pediatric hypertension was observed in those with a higher developmental stage (*p* = 0.046) (Figure 2b). In addition, the obese children had a significantly higher rate of hypertension than the non-obese children (*p* < 0.001) (Figure 2c). There was a trend of an increasing prevalence of hypertension in the children with more severe OSAS (*p* < 0.001) (Figure 2d). Moreover, the children with abnormal SpO_2_ or significant hypoxemia had significantly higher rates of hypertension than those with normal SpO_2_ or non-significant hypoxemia (*p* = 0.001 and 0.013, respectively) (Figure 2e,f, respectively). Furthermore, the differences in the positive rates of family history of hypertension, OSAS, and obesity between the hypertensive and normotensive subgroups did not reach statistical significance (*p* = 0.221, 0.680, and 0.125, respectively).

### 3.3. Associations between Systolic Blood Pressure, Diastolic Blood Pressure, and Clinical Variables

**Figure 3:**
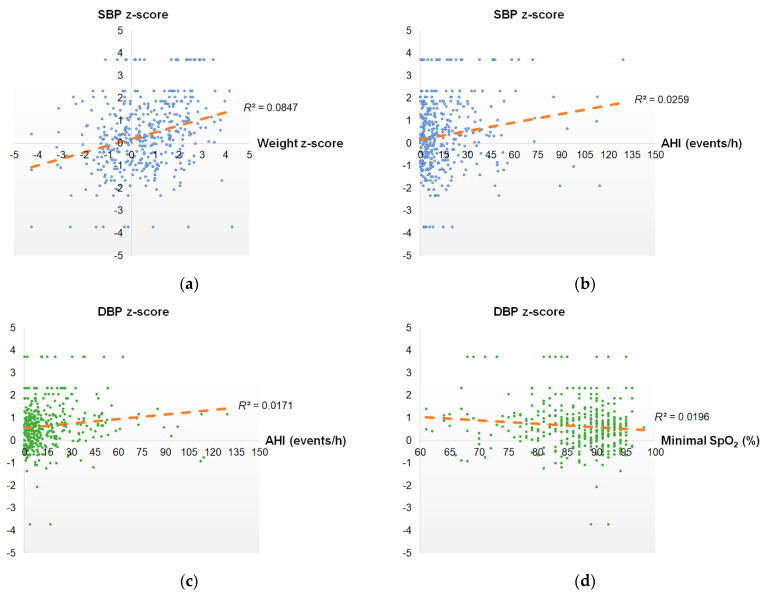
Correlation charts demonstrate the relationships between blood pressure and selected clinical variables. (**a**) Systolic blood pressure z-score versus weight z-score; (**b**) Systolic blood pressure z-score versus apnea-hypopnea index; (**c**) Diastolic blood pressure z-score versus apnea-hypopnea index; (**d**) Diastolic blood pressure z-score versus minimal oxygen saturation. The data are analyzed using Pearson correlation test. Abbreviations: AHI: apnea-hypopnea index; DBP: diastolic blood pressure; SpO_2_: oxygen saturation; SBP: systolic blood pressure.

SBP z-score was significantly associated with weight z-score (*r* = 0.29, *p* < 0.001) (Figure 3a), BMI z-score (*r* = 0.29, *p* < 0.001) (Figure 3b), AHI (*r* = 0.16, *p* = 0.001), mean SpO_2_ (*r* = −0.12, *p* = 0.018), and minimal SpO_2_ (*r* = −0.15, *p* = 0.002). To improve the regression performance, BMI z-score was removed due to overlapping information. Therefore, the full model including weight z-score, AHI, mean SpO_2_, and minimal SpO_2_ was further analyzed using multivariate linear regression analysis (adjusted *R*^2^ = 0.095) (Table 2). Weight z-score (regression coefficient (B) = 0.27, 95% confidence interval (CI) = 0.17–0.37) was significantly associated with SBP z-score under the control of AHI, mean SpO_2_ and minimal SpO_2_. Using a forward selection approach to obtain the parsimonious model, weight z-score (B = 0.27, 95% CI = 0.18–0.37) and AHI (B = 0.01, 95% CI = 0.001–0.02) were significantly and independently correlated with SBP z-score (*R*^2^ = 0.090).

DBP z-score was significantly related to weight z-score (*r* = 0.12, *p* = 0.020), BMI z-score (*r* = 0.13, *p* = 0.011), AHI (*r* = 0.13, *p* = 0.009) (Figure 3c), and minimal SpO_2_ (*r* = −0.14, *p* = 0.005) (Figure 3d). However, BMI z-score was removed due to overlapping information, and mean SpO_2_ was removed due to an incorrect sign of regression coefficient. Nevertheless, none of the variables significantly predicted DBP z-score using the full model (weight z-score, AHI, and minimal SpO_2_) (*R*^2^ = 0.029). The parsimonious model including minimal SpO_2_ (B = −0.02, 95% CI = −0.03–−0.01) was significantly and independently correlated with SBP z-score (*R*^2^ = 0.020).

### 3.4. Variables for Predicting Pediatric Hypertension

Using Spearman’s correlation test, pediatric hypertension was associated with male sex (*r* = 0.11, *p* = 0.028), age (*r* = 0.07, *p* = 0.15), developmental stage (*r* = 0.10, *p* = 0.052), weight z-score (*r* = 0.21, *p* < 0.001), BMI z-score (*r* = 0.21, *p* < 0.001), obesity (*r* = 0.24, *p* < 0.001), AHI (*r* = 0.18, *p* = 0.001), disease severity (*r* = 0.18, *p* < 0.001), mean SpO_2_ (*r* = −0.15, *p* = 0.003), abnormal SpO_2_ (*r* = 0.17, *p* = 0.001), minimal SpO_2_ (*r* = −0.13, *p* = 0.012), and significant hypoxemia (*r* = 0.13, *p* = 0.013). In further logistic regression analyses, age, weight z-score, BMI z-score, AHI, mean SpO_2_, and minimal SpO_2_ were removed because of overlapping information.

Table 3 demonstrates the univariate logistic regression models to predict pediatric hypertension in the overall cohort. Notably, male sex (odds ratio (OR) = 1.80; 95% CI = 1.06–3.07), obesity (OR = 3.09, 95% CI = 1.93–4.96), abnormal SpO_2_ (OR = 2.40; 95% CI = 1.41–4.08), and significant hypoxemia (OR = 1.78; 95% CI = 1.13–2.82) significantly predicted hypertension. Compared to the toddler/preschooler group, the late childhood/adolescent group had an increased odds of hypertension (OR = 1.74; 95% CI = 1.02–2.97). Severe OSAS had higher odds of hypertension (OR = 2.35; 95% CI = 1.41–3.92) than mild OSAS. Therefore, late childhood/adolescence and severe OSAS were included in the full models. Otherwise, family histories of hypertension, OSAS, and obesity did not significantly predict pediatric hypertension (*p* = 0.223, 0.456, and 0.123, respectively).

Significant hypoxemia was removed from the full models due to incorrect sign of regression coefficient. Table 4 presents the multivariate logistic regression analyses for predicting pediatric hypertension in the overall cohort. The final full model (*R*^2^ = 0.155) included male sex (OR = 1.60, 95% CI = 0.91–2.82), late childhood/adolescence (OR = 1.67, 95% CI = 1.02–2.74), obesity (OR = 2.48, 95% CI = 1.51–4.07), severe OSAS (OR = 1.99, 95% CI = 1.20–3.32), and abnormal SpO_2_ (OR = 1.70, 95% CI = 0.93–3.11). Using a forward selection approach, the parsimonious model included late childhood/adolescence (OR = 1.72, 95% CI = 1.05–2.81, *p* = 0.031), obesity (OR = 2.58, 95% CI = 1.58–4.22, *p* < 0.001), and severe OSAS (OR = 2.38, 95% CI = 1.48–3.81, *p* < 0.001) (*R*^2^ = 0.139).

### 3.5. Variables Independently Associated with Hypertension in Obese and Non-Obese Children with OSAS Using Logistic Regression Analysis

For the obese children (*n* = 111), the full model (*R*^2^ = 0.209) included male sex (OR = 1.78, 95% CI = 0.58–5.41, *p* = 0.315), late childhood/adolescence (OR = 2.39, 95% CI = 1.03–5.56, *p* = 0.044), severe OSAS (OR = 1.59, 95% CI = 0.65–3.92, *p* = 0.313), and abnormal SpO_2_ (OR = 4.42, 95% CI = 1.42–13.75, *p* = 0.010). Furthermore, the parsimonious model included late childhood/adolescence (OR = 2.50, 95% CI = 1.10–5.71, *p* = 0.030) and abnormal SpO_2_ (OR = 4.91, 95% CI = 1.81–13.27, *p* = 0.002) (R^2^ = 0.185). The prevalence of hypertension in the obese children with abnormal SpO_2_ in the late childhood/adolescent group was significantly higher than that in the obese children with normal SpO_2_ in the toddler/preschooler/early childhood group (57.9% vs. 29.6%, *p* = 0.003).

For the non-obese children (*n* = 280), the full model (*R*^2^ = 0.055) included male sex (OR = 1.58, 95% CI = 0.80–3.09, *p* = 0.185), late childhood/adolescence (OR = 1.35, 95% CI = 0.71–2.57, *p* = 0.363), severe OSAS (OR = 2.18, 95% CI = 1.17–4.05, *p* = 0.014), and abnormal SpO_2_ (OR = 1.04, 95% CI = 0.48–2.28, *p* = 0.916). Severe OSAS (OR = 2.28, 95% CI = 1.27–4.10, *p* = 0.006) was an independent predictor in the parsimonious model (*R*^2^ = 0.046). The prevalence of hypertension in the non-obese children with severe OSAS was significantly higher than that in the non-obese children without severe OSAS (29.0% vs. 15.2%, *p* = 0.005).

## 4. Discussion

Children with hypertension are prone to a cluster of cardio-metabolic risks [36], and BP in childhood is highly predictive of BP in adulthood [9]. Early interventions are recommended to attain optimal BP control through multidisciplinary approaches, such as lifestyle modification, weight reduction, or anti-hypertensive drugs [36]. The estimated prevalence of pediatric hypertension varies widely, ranging from 0.8% to 13.3% [9,37]. However, in this sample of children with OSAS, the prevalence of hypertension was alarmingly higher, 27.1% for the overall cohort and 43.9% for the obese patients. In addition, weight z-score and AHI were independently associated with SBP z-score, and minimal SpO_2_ was independently related to DBP z-score. Furthermore, late childhood/adolescence, obesity, and severe OSAS were independently associated with hypertension. After adjusting SDP z-score, DBP z-score, and hypertension for sex, age, and height referenced to a public database, developmental stage and obesity were still significantly associated with hypertension. Our findings support that risk stratification and a timely diagnosis are more crucial in this particular group than in the general population for the initiation of treatment [38].

Obesity is the most well-documented risk factor for pediatric hypertension [36,39], which is consistent with our results that the hypertensive group had a much higher BMI z-score. In addition, weight z-score was independently correlated with SBP z-score, and in the overall cohort, obesity independently predicted hypertension. However, in addition to the known associations between obesity and elevated BP, there were also several novel and interesting observations in the present study. Not only was the prevalence of hypertension higher in the obese children with OSAS, but there were also disparities in the risk factors for hypertension between patients with and without obesity.

In this study, demographic variables were associated with BP. Some previous investigations have shown a sex difference in the prevalence of pediatric hypertension. For example, the rates of pre-hypertension and first stage systemic hypertension were observed to be two-fold higher in boys than in girls among school children in a study from Western Ukraine [37], and a systematic review found that boys were more likely to have high BP than girls [40]. In the current study, there was a significantly larger proportion of boys in the hypertensive group than in the normotensive group. In addition, male sex was positively associated with elevated SBP and pediatric hypertension. However, the effect of sex became insignificant after adjusting for other variables.

On the other hand, the role of age emerged after the adjustments, and the effect differed with weight status. Late childhood/adolescence predicted pediatric hypertension in the overall cohort, which is consistent with previous studies. Díaz and Calandra reported that high BP was more prevalent among adolescents than children ≤10 years old [40]. A large cohort study also showed that increasing age was significantly associated with pre-hypertension and confirmed hypertension [40]. Notably, hypertension seemed to be more common in the obese children than in the non-obese children in all age groups, although the difference in prevalence reached statistical significance only after late childhood. Moreover, for obese children with OSAS, late childhood/adolescence predicted hypertension, while age was not an independent predictor for non-obese children. In a 10-year follow-up study, Chan et al. reported that 30% of pediatric OSA cases resolved after a decade, and that the 22% who continued to have OSA were predominantly male and had a higher BMI z-score [41]. OSAS, as a chronic condition, has long-term accumulative detrimental effects leading to adverse health outcomes later in life [38]. The bidirectional pathogenic force of obesity and OSAS could worsen over time and exacerbate the disease itself or comorbidities. The disease burden of OSAS may worsen over time, especially in children with obesity.

In this study, we also observed significant associations between OSAS severity and BP. AHI was independently associated with SBP z-score. Furthermore, disease severity was higher in the hypertensive group than in the normotensive group. In the overall cohort, OSAS severity independently predicted pediatric hypertension. However, subgroup analysis showed that the effects of OSAS severity were different for the patients with different weight status. In the non-obese children, OSAS severity was the only predictor of hypertension, whereas, in the obese children, OSAS severity was not an independent predictor. In an earlier study of ours, we found that non-obese pediatric OSA patients had higher levels of inflammation than those who were obese, and inflammation increased with disease severity [22]. Accordingly, we suggest that for pediatric OSAS patients who are not obese, systemic inflammation may play a central role in the development and presentation of comorbidities.

Desaturation, a hallmark manifestation of OSAS, was assessed with mean SpO_2_ and minimal SpO_2_ in this study. Mean SpO_2_ represents the patients’ average oxygen saturation during sleep, while minimal SpO_2_ represents the nadir. We found that minimal SpO_2_ had a closer correlation with BP than mean SpO_2_. Nevertheless, abnormal SpO_2_ became an independent predictor for hypertension in the obese subgroup, but not in the non-obese subgroup. Intermittent hypoxemia is a well-known pathophysiological mechanism caused by OSA [42]. Animal models and clinical data from humans have suggested that intermittent hypoxemia induces oxidative stress and sympathetic hyperactivity, which consequently lead to hemodynamic alterations and vascular dysfunction [38,42]. It is possible that the negative effect of hypoxemia on BP worsens when co-existing with obesity. Future research is warranted to better understand the impact of hypoxemia on BP elevation and the role of obesity in the pathogenic process.

Noteworthily, the associations of hypertension with age, obesity, OSAS, and ethnicity are complex. For example, Asian children have a higher blood pressure compared to White children [43]. Moreover, the definition of obesity differs across ethnicities [44]. For example, Asian children tend to have a lower BMI compared to the WHO reference [45] and have 3–6 units lower BMI given the same percentage body fat compared with Caucasians [46]. Therefore, ethnic differences in hypertension and obesity should be considered when developing population-based programs and policies. Family history may also play an essential role in pediatric hypertension in the general population. However, the heritable associations of pediatric hypertension with family histories of hypertension, OSAS, and obesity were not statistically significant in children with OSAS in this study.

Some previous studies suggest a strong relationship between hypertension, OSA, and the cardiac autonomic nervous system [47,48]. The electrocardiogram-detected heart rate variability is widely used to monitor cardiac autonomic function for many years [49]. Recently, the analyses of electrogram have been used to detect OSA and hypertension [50,51,52]. For example, the information-based similarity of the regularity of heart rate fluctuations between adjacent RR segments decreased significantly only in the severe OSA group. However, this association was not significant in the non-severe OSA group [53]. These findings suggested that only severe OSAS have an important effect on cardiac autonomic function and possibly explained our finding in non-obese children with OSAS.

In the current study, we identified relationships between demographic variables, weight status, breathing disorder parameters, and BP in a sample of children with OSAS, and there were differences in the predictors of pediatric hypertension between the patients with and without obesity. Many doctors may be unaware of the increased risk of hypertension among pediatric OSA patients without obesity [54], and the current study further highlights the association between hypertension and severe OSAS in this population. Previous studies have reported a reduction in BP after adenotonsillectomy in pediatric OSA patients [55,56], and weight reduction has been reported to be an effective approach to obesity-related pediatric hypertension [57,58]. To prevent the development and aggravation of hypertension, risk factors, including severe OSAS, hypoxemia, and obesity, should be addressed individually and in a timely manner. Notably, there has been an 80% decrease in the diagnosis and management of OSAS worldwide during the COVID−19 pandemic [59]. A 3% oxygen desaturation index ≥6.0 events/h using a home sleep pulse oximeter can predict severe disease in OSAS children with an acceptable accuracy of 82% [60]. Of note, mean SpO_2_ is also measured by pulse oximetry. Our findings support the use of home sleep pulse oximetry as an alternative measure of standard in-laboratory polysomnography, particularly during the COVID-19 pandemic.

There were some limitations to this study. First, due to the cross-sectional design, we could not assess causal relationships. A recent longitudinal study has revealed that childhood OSA was associated with adolescent hypertension, whereas a remitted OSA was not [61]. These findings suggest causality existing between OSA and hypertension in children. Second, the investigation was conducted at a single medical center in Taiwan, and most of the participants were of Han ancestry, which may limit the generalizability of our findings. Third, the inclusion of dichotomized variables in the multivariable model may have reduced the statistical power and resulted in valuable information being lost [61]. To improve the classification and predictive accuracy [62], we used several statistical methods, including full models, external information, the sign of regression coefficients, and a forward selection approach [32]. However, the risk of losing valuable information still existed. Although this study suggests some important predictors for pediatric hypertension in children with OSAS, further studies are warranted to assess traditional hypertension risk factors, such as high sodium intake and different ethnicities.

## 5. Conclusions

This study highlights the importance of end-organ dysfunction in OSA and how multiple factors significantly contribute to the morbidity and possible mortality of children affected by OSAS, hypertension, and obesity. Late childhood/adolescence, obesity, and severe OSAS were independently and significantly associated with hypertension among pediatric OSAS patients. However, the risk factors differed according to weight status. Disease severity was the strongest predictor of hypertension in the non-obese children, while late childhood/adolescence and abnormal SpO_2_ were more influential in those who were obese. Children with OSAS should be carefully screened for hypertension, and the timing and approach of interventions should take weight status into consideration.

## Figures and Tables

**Table 1:** Comparisons of clinical variables between the hypertensive and normotensive groups.

Variables	Hypertensive Group	Normotensive Group	*p*-Value
Patients	*n* = 107	*n* = 289	
Blood pressure measures			
SBP z-score	2.019 (1.258)	−0.250 (1.102)	<0.001 ^1^
DBP z-score	1.554 (1.178)	0.317 (0.671)	<0.001 ^1^
Demographic measures			
Sex			
Female, *n* (%)	22 (19.3)	92 (80.7)	0.028 ^2^
Male, *n* (%)	85 (30.1)	197 (69.9)	
Age, years	7.88 (3.01)	7.36 (2.60)	0.115 ^1^
Development stage			
Toddler/preschooler, *n* (%)	35 (24.8)	106 (75.2)	0.046 ^3^
Early childhood, *n* (%)	29 (21.2)	108 (78.8)	
Late childhood/adolescence, *n* (%)	43 (36.4)	75 (63.6)	
Anthropometric measures			
Weight z-score	1.066 (1.486)	0.362 (1.458)	<0.001 ^1^
Height z-score	0.345 (1.133)	0.102 (1.166)	0.064 ^1^
BMI z-score	1.088 (1.459)	0.429 (1.434)	<0.001 ^1^
Obesity			
Non-obesity, *n* (%)	58 (20.4)	227 (79.6)	<0.001 ^2^
Obesity, *n* (%)	62 (55.9)	49 (44.1)	
Polysomnographic measures			
AHI, events/h	20.59 (21.50)	13.37 (17.72)	0.003 ^1^
Disease severity			
Mild OSAS, *n* (%)	29 (20.9)	110 (79.1)	<0.001 ^3^
Moderate OSAS, *n* (%)	13 (14.9)	74 (85.1)	
Severe OSAS, *n* (%)	65 (38.2)	105 (61.8)	
Mean SpO_2_, %	95.15 (3.18)	96.01 (2.85)	0.015 ^1^
Abnormal SpO_2_			
Normal SpO_2_, *n* (%)	76 (23.5)	247 (76.5)	0.001 ^2^
Abnormal SpO_2_, *n* (%)	31 (42.5)	42 (57.5)	
Minimal SpO_2_, %	83.02 (11.39)	86.32 (8.00)	0.007 ^1^
Significant hypoxemia			
Non-significant hypoxemia, *n* (%)	61 (23.1)	203 (76.9)	0.013 ^2^
Significant hypoxemia, *n* (%)	46 (34.8)	86 (65.2)	
Family history of hypertension			
Absence, *n* (%)	89 (25.9)	254 (74.1)	0.221 ^2^
Presence, *n* (%)	18 (34.0)	35 (66.0)	
Family history of OSAS			
Absence, *n* (%)	106 (27.2)	283 (72.8)	0.680 ^4^
Presence, *n* (%)	1 (14.3)	6 (85.7)	
Family history of obesity			
Absence, *n* (%)	104 (26.6)	287 (73.4)	0.125 ^4^
Presence, *n* (%)	3 (60.0)	2 (40.0)	

Data are summarized as mean (standard deviation) or *n* (%) as appropriate. Abbreviations: AHI: apnea-hypopnea index; BMI: body mass index; DBP: diastolic blood pressure; OSAS: obstructive sleep apnea syndrome; SBP: systolic blood pressure; SpO_2_: oxygen saturation. ^1^ Data were compared using the independent-samples *t*-test. ^2^ Data were compared using the chi-square test. ^3^ Data were compared using the Mantel-Haenszel test for trend. ^4^ Data were compared using the Fisher’s exact test.

**Table 2:** Regression models of systolic and diastolic blood pressure in the overall cohort.

Predictors	Regression Coefficient (95% CI)	*p*-Value ^1^	Regression Coefficient (95% CI)	*p*-Value ^1^
	Full model		Parsimonious model	
Systolic blood pressure z-score				
Weight z-score	0.27 (0.17–0.37)	<0.001	0.27 (0.18–0.37)	<0.001
AHI, events/h	0.01 (−0.01–0.02)	0.314	0.01 (0.001–0.02)	0.043
Mean SpO_2_, %	−0.002 (−0.07–0.06)	0.959		NI ^2^
Minimal SpO_2_, %	−0.01 (−0.03–0.02)	0.505		NI ^2^
Model summary	*R*^2^ = 0.095		*R*^2^ = 0.090	
Diastolic blood pressure z-score				
Weight z-score	0.06 (−0.01–0.12)	0.100		NI ^2^
AHI, events/h	0.003 (−0.004–0.01)	0.401		NI ^2^
Minimal SpO_2_, %	−0.01 (−0.02–0.01)	0.210	−0.02 (−0.03–−0.01)	0.005
Model summary	*R*^2^ = 0.029		*R*^2^ = 0.020	

Abbreviations: AHI: apnea-hypopnea index; CI: confidence interval; NI: not included; SpO_2_: oxygen saturation. ^1^ Data were compared using linear regression models. ^2^ The variable was not included after forward selection in the parsimonious model.

**Table 3:** Univariate logistic regression models for predicting pediatric hypertension in the overall cohort.

Predictors	Odds Ratio (95%CI)	*p*-Value ^1^
Sex		
Female	reference	
Male	1.80 (1.06–3.07)	0.029
Developmental stage		
Toddler/preschooler	reference	
Early childhood	0.81 (0.46–1.42)	0.470
Late childhood/adolescence	1.74 (1.02–2.97)	0.043
Obesity		
Non-obesity	reference	
Obesity	3.09 (1.93–4.96)	<0.001
Disease severity		
Mild OSAS	reference	
Moderate OSAS	0.67 (0.33–1.37)	0.267
Severe OSAS	2.35 (1.41–3.92)	0.001
Abnormal SpO_2_		
Normal SpO_2_	reference	
Abnormal SpO_2_	2.40 (1.41–4.08)	0.001
Significant hypoxemia		
Non-significant hypoxemia	reference	
Significant hypoxemia	1.78 (1.13–2.82)	0.014
Family history of hypertension		
Absence	reference	
Presence	1.47 (0.79–2.72)	0.223
Family history of OSAS		
Absence	reference	
Presence	0.45 (0.05–3.74)	0.456
Family history of obesity		
Absence	reference	
Presence	4.14 (0.68–25.1)	0.123

Abbreviations: CI: confidence interval; OSAS: obstructive sleep apnea syndrome; SpO_2_: oxygen saturation. ^1^ Data were compared using univariate logistic regression models.

**Table 4:** Multivariate logistic regression analyses for predicting pediatric hypertension in the overall cohort.

Predictors	Odds Ratio (95%CI)	*p*-Value ^1^	Odds Ratio (95%CI)	*p*-Value ^1^
	Full model		Parsimonious model	
Female sex	reference			
Male sex	1.61 (0.91–2.82)	0.101		NI ^2^
Toddler/preschooler/early childhood	reference		reference	
Late childhood/adolescence	1.67 (1.02–2.74)	0.044	1.72 (1.05–2.81)	0.031
Non-obesity	reference		reference	
Obesity	2.58 (1.51–4.07)	<0.001	2.58 (1.58–4.22)	<0.001
Non-severe OSAS	reference		reference	
Severe OSAS	1.99 (1.20–3.32)	0.008	2.38 (1.48–3.81)	<0.001
Normal SpO_2_	reference			
Abnormal SpO_2_	1.70 (0.93–3.11)	0.084		NI ^2^

Abbreviations: CI: confidence interval; NI: not included; OSAS: obstructive sleep apnea syndrome; SpO_2_: oxygen saturation. ^1^ Data were compared using multivariate logistic regression models. ^2^ The variable was not included after forward selection in the parsimonious model.

## Data Availability

The data presented in this study are available on request from the corresponding author. The data are not publicly available due to ethical restrictions.

## References

[1] Lumeng J.C., Chervin R.D. (2008). Epidemiology of pediatric obstructive sleep apnea. Proc. Am. Thorac. Soc..

[2] Li H.Y., Lee L.A. (2009). Sleep-disordered breathing in children. Chang. Gung. Med. J..

[3] O’Brien L.M., Mervis C.B., Holbrook C.R., Bruner J.L., Klaus C.J., Rutherford J., Raffield T.J., Gozal D. (2004). Neurobehavioral implications of habitual snoring in children. Pediatrics.

[4] Katz E.S., D’Ambrosio C.M. (2008). Pathophysiology of pediatric obstructive sleep apnea. Proc. Am. Thorac. Soc..

[5] Chuang H.H., Hsu J.F., Chuang L.P., Chen N.H., Huang Y.S., Li H.Y., Chen J.Y., Lee L.A., Huang C.G. (2020). Differences in Anthropometric and Clinical Features among Preschoolers, School-Age Children, and Adolescents with Obstructive Sleep Apnea-A Hospital-Based Study in Taiwan. Int. J. Environ. Res. Publ. Health.

[6] Jackman A.R., Biggs S.N., Walter L.M., Embuldeniya U.S., Davey M.J., Nixon G.M., Anderson V., Trinder J., Horne R.S. (2012). Sleep-disordered breathing in preschool children is associated with behavioral, but not cognitive, impairments. Sleep Med..

[7] Horne R.S., Yang J.S., Walter L.M., Richardson H.L., O’Driscoll D.M., Foster A.M., Wong S., Ng M.L., Bashir F., Patterson R. (2011). Elevated blood pressure during sleep and wake in children with sleep-disordered breathing. Pediatrics.

[8] Song P., Zhang Y., Yu J., Zha M., Zhu Y., Rahimi K., Rudan I. (2019). Global Prevalence of Hypertension in Children: A Systematic Review and Meta-analysis. JAMA Pediatr..

[9] Rao G. (2016). Diagnosis, Epidemiology, and Management of Hypertension in Children. Pediatrics.

[10] Hardy S.T., Urbina E.M. (2021). Blood Pressure in Childhood and Adolescence. Am. J. Hypertens.

[11] Young T., Peppard P.E., Gottlieb D.J. (2002). Epidemiology of obstructive sleep apnea: A population health perspective. Am. J. Respir. Crit. Care Med..

[12] Nisbet L.C., Yiallourou S.R., Walter L.M., Horne R.S. (2014). Blood pressure regulation, autonomic control and sleep disordered breathing in children. Sleep Med. Rev..

[13] Brooks D.M., Kelly A., Sorkin J.D., Koren D., Chng S.Y., Gallagher P.R., Amin R., Dougherty S., Guo R., Marcus C.L. (2020). The relationship between sleep-disordered breathing, blood pressure, and urinary cortisol and catecholamines in children. J. Clin. Sleep Med..

[14] Gonzaga C., Bertolami A., Bertolami M., Amodeo C., Calhoun D. (2015). Obstructive sleep apnea, hypertension and cardiovascular diseases. J. Hum. Hypertens.

[15] Bixler E.O., Vgontzas A.N., Lin H.M., Liao D., Calhoun S., Fedok F., Vlasic V., Graff G. (2008). Blood pressure associated with sleep-disordered breathing in a population sample of children. Hypertension.

[16] Archbold K.H., Vasquez M.M., Goodwin J.L., Quan S.F. (2012). Effects of sleep patterns and obesity on increases in blood pressure in a 5-year period: Report from the Tucson Children’s Assessment of Sleep Apnea Study. J. Pediatr..

[17] Zintzaras E., Kaditis A.G. (2007). Sleep-disordered breathing and blood pressure in children: A meta-analysis. Arch Pediatr. Adolesc Med..

[18] Tagetti A., Bonafini S., Zaffanello M., Benetti M.V., Vedove F.D., Gasperi E., Cavarzere P., Gaudino R., Piacentini G., Minuz P. (2017). Sleep-disordered breathing is associated with blood pressure and carotid arterial stiffness in obese children. J. Hypertens.

[19] Kang K.T., Lee P.L., Weng W.C., Hsu W.C. (2012). Body weight status and obstructive sleep apnea in children. Int. J. Obes. (Lond).

[20] Walter L.M., Tamanyan K., Limawan A.P., Biggs S.N., Weichard A.J., Davey M.J., Nixon G.M., Horne R.S.C. (2018). Overweight and obese children with sleep disordered breathing have elevated arterial stiffness. Sleep Med..

[21] Evans C.A., Selvadurai H., Baur L.A., Waters K.A. (2014). Effects of obstructive sleep apnea and obesity on exercise function in children. Sleep.

[22] Chuang H.H., Huang C.G., Chuang L.P., Huang Y.S., Chen N.H., Li H.Y., Fang T.J., Hsu J.F., Lai H.C., Chen J.Y. (2020). Relationships Among and Predictive Values of Obesity, Inflammation Markers, and Disease Severity in Pediatric Patients with Obstructive Sleep Apnea Before and After Adenotonsillectomy. J. Clin. Med..

[23] von Elm E., Altman D.G., Egger M., Pocock S.J., Gotzsche P.C., Vandenbroucke J.P., Initiative S. (2007). The Strengthening the Reporting of Observational Studies in Epidemiology (STROBE) statement: Guidelines for reporting observational studies. PLoS Med..

[24] Gottlieb D.J., Vezina R.M., Chase C., Lesko S.M., Heeren T.C., Weese-Mayer D.E., Auerbach S.H., Corwin M.J. (2003). Symptoms of sleep-disordered breathing in 5-year-old children are associated with sleepiness and problem behaviors. Pediatrics.

[25] Huang Y.S., Guilleminault C., Hwang F.M., Cheng C., Lin C.H., Li H.Y., Lee L.A. (2016). Inflammatory cytokines in pediatric obstructive sleep apnea. Medicine.

[26] Flegal K.M., Cole T.J. (2013). Construction of LMS parameters for the Centers for Disease Control and Prevention 2000 growth charts. Nat. Health Stat. Rep..

[27] Kuczmarski R.J., Ogden C.L., Guo S.S., Grummer-Strawn L.M., Flegal K.M., Mei Z., Wei R., Curtin L.R., Roche A.F., Johnson C.L. (2002). 2000 CDC Growth Charts for the United States: Methods and development. Vit. Health Stat..

[28] Huang Y.S., Guilleminault C., Lee L.A., Lin C.H., Hwang F.M. (2014). Treatment outcomes of adenotonsillectomy for children with obstructive sleep apnea: A prospective longitudinal study. Sleep.

[29] Berry R.B., Budhiraja R., Gottlieb D.J., Gozal D., Iber C., Kapur V.K., Marcus C.L., Mehra R., Parthasarathy S., Quan S.F. (2012). Rules for scoring respiratory events in sleep: Update of the 2007 AASM Manual for the Scoring of Sleep and Associated Events. Deliberations of the Sleep Apnea Definitions Task Force of the American Academy of Sleep Medicine. J. Clin. Sleep Med. JCSM Off. Publ. Am. Acad. Sleep Med..

[30] National High Blood Pressure Education Program Working Group on High Blood Pressure in Children and Adolescents (2004). The fourth report on the diagnosis, evaluation, and treatment of high blood pressure in children and adolescents. Pediatrics.

[31] Flynn J.T., Kaelber D.C., Baker-Smith C.M., Blowey D., Carroll A.E., Daniels S.R., de Ferranti S.D., Dionne J.M., Falkner B., Flinn S.K. (2017). Clinical Practice Guideline for Screening and Management of High Blood Pressure in Children and Adolescents. Pediatrics.

[32] Steyerberg E.W., Eijkemans M.J.C., Harrell F.E., Habbema J.D.F. (2000). Prognostic modelling with logistic regression analysis: A comparison of selection and estimation methods in small data sets. Stat. Med..

[33] Chang I.S., Kang K.T., Tseng C.C., Weng W.C., Hsiao T.Y., Lee P.L., Hsu W.C. (2018). Revisits after adenotonsillectomy in children with sleep-disordered breathing: A retrospective single-institution study. Clin. Otolaryngol..

[34] Dehlink E., Tan H.L. (2016). Update on paediatric obstructive sleep apnoea. J. Thorac. Dis..

[35] Brown K.A., Laferriere A., Lakheeram I., Moss I.R. (2006). Recurrent hypoxemia in children is associated with increased analgesic sensitivity to opiates. Anesthesiology.

[36] Wühl E. (2019). Hypertension in childhood obesity. Acta Paediatr..

[37] Pavlyshyn H., Furdela V., Kovalchuk T., Haliyash N., Luchyshyn N. (2017). Epidemiological aspects of obesity and systemic hypertension amongschool children of Western Ukraine. Pediatr. Endocrinol. Diabetes Metab..

[38] Horne R.S.C. (2020). Endothelial Damage in Children with Sleep-disordered Breathing. Am. J. Respir. Crit. Care Med..

[39] Hsu C.Y., Lin R.H., Lin Y.C., Chen J.Y., Li W.C., Lee L.A., Liu K.H., Chuang H.H. (2020). Are Body Composition Parameters Better than Conventional Anthropometric Measures in Predicting Pediatric Hypertension?. Int. J. Environ. Res. Public Health.

[40] Díaz A., Calandra L. (2017). High blood pressure in school children and adolescents in Argentina over the past 25 years: A systematic review of observational studies. Arch Argent Pediatr..

[41] Chan K.C., Au C.T., Hui L.L., Ng S.K., Wing Y.K., Li A.M. (2019). How OSA Evolves From Childhood to Young Adulthood: Natural History From a 10-Year Follow-up Study. Chest.

[42] Kimura H., Ota H., Kimura Y., Takasawa S. (2019). Effects of Intermittent Hypoxia on Pulmonary Vascular and Systemic Diseases. Int. J. Environ. Res. Publ. Health.

[43] Battu H.S., Bhopal R., Agyemang C. (2018). Heterogeneity in blood pressure in UK Bangladeshi, Indian and Pakistani, compared to White, populations: Divergence of adults and children. J. Hum. Hyper..

[44] Qian K., Tan L., Li S., Li Z., Yu F., Liang H., Gao S., Ren X., Zhang J., Zhang Z. (2020). Comparison of different BMI cut-offs to screen for child and adolescent obesity in urban China. Publ. Health Nutr..

[45] de Wilde J.A., van Dommelen P., Middelkoop B.J. (2013). Appropriate body mass index cut-offs to determine thinness, overweight and obesity in South Asian children in the Netherlands. PLoS ONE.

[46] Liu A., Byrne N.M., Kagawa M., Ma G., Poh B.K., Ismail M.N., Kijboonchoo K., Nasreddine L., Trinidad T.P., Hills A.P. (2011). Ethnic differences in the relationship between body mass index and percentage body fat among Asian children from different backgrounds. Br. J. Nutr..

[47] Cai A., Wang L., Zhou Y. (2016). Hypertension and obstructive sleep apnea. Hyper. Res..

[48] May A.M., Van Wagoner D.R., Mehra R. (2017). OSA and Cardiac Arrhythmogenesis: Mechanistic Insights. Chest.

[49] Kleiger R.E., Stein P.K., Bigger J.T. (2005). Heart rate variability: Measurement and clinical utility. Ann. Noninv. Electrocardiol..

[50] Fan X., Wang H., Xu F., Zhao Y., Tsui K.-L. (2020). Homecare-Oriented Intelligent Long-Term Monitoring of Blood Pressure Using Electrocardiogram Signals. IEEE Trans. Ind. Inf..

[51] Shen Q., Qin H., Wei K., Liu G. (2021). Multiscale Deep Neural Network for Obstructive Sleep Apnea Detection Using RR Interval From Single-Lead ECG Signal. IEEE Trans. Inst. Meas..

[52] de Chazal P., Penzel T., Heneghan C. (2004). Automated detection of obstructive sleep apnoea at different time scales using the electrocardiogram. Physiol. Meas..

[53] Wu S., Liang D., Yang Q., Liu G. (2021). Regularity of heart rate fluctuations analysis in obstructive sleep apnea patients using information-based similarity. Biomed. Sig. Proc. Control.

[54] Hussain S.F., Zahid S., Haqqee R., Khan J.A. (2003). General physicians’ perspective of sleep apnea from a developing country. Southeast Asian J. Trop. Med. Public Health.

[55] Lee L.A., Li H.Y., Lin Y.S., Fang T.J., Huang Y.S., Hsu J.F., Wu C.M., Huang C.G. (2015). Severity of childhood obstructive sleep apnea and hypertension improved after adenotonsillectomy. Otolaryngol. Head Neck Surg..

[56] Lee C.H., Kang K.T., Chiu S.N., Chang I.S., Weng W.C., Lee P.L., Hsu W.C. (2018). Association of Adenotonsillectomy With Blood Pressure Among Hypertensive and Nonhypertensive Children With Obstructive Sleep Apnea. JAMA Otolaryngol. Head Neck Surg..

[57] Brady T.M. (2017). Obesity-Related Hypertension in Children. Front. Pediatr..

[58] Hagman E., Danielsson P., Elimam A., Marcus C. (2019). The effect of weight loss and weight gain on blood pressure in children and adolescents with obesity. Int. J. Obes..

[59] Grote L., McNicholas W.T., Hedner J., Collaborators E. (2020). Sleep apnoea management in Europe during the COVID-19 pandemic: Data from the European Sleep Apnoea Database (ESADA). Eur. Respir J..

[60] Hsieh H.S., Kang C.J., Chuang H.H., Zhuo M.Y., Lee G.S., Huang Y.S., Chuang L.P., Kuo T.B.J., Yang C.C.H., Lee L.A. (2021). Screening Severe Obstructive Sleep Apnea in Children with Snoring. Diagnostics.

[61] Altman D.G., Royston P. (2006). The cost of dichotomising continuous variables. BMJ.

[62] Pepe M.S., Janes H., Longton G., Leisenring W., Newcomb P. (2004). Limitations of the odds ratio in gauging the performance of a diagnostic, prognostic, or screening marker. Am. J. Epidemiol..

